# Early kinetics of serum amyloid A predict clinical benefit to first-line chemoimmunotherapy and immunotherapy in advanced non-small cell lung cancer: a retrospective analysis

**DOI:** 10.1186/s40364-025-00791-1

**Published:** 2025-05-24

**Authors:** Wei Du, Jianhua Zhan, Kai Wu, Yanming Wang, Li Zhang, Shaodong Hong

**Affiliations:** 1 State Key Laboratory of Oncology in South China, Guangdong Provincial Clinical Research Center for Cancer, Sun Yat-Sen University Cancer Center, Guangzhou, 510060 People’s Republic of China; 2 Department of Medical Oncology, Sun Yat-Sen University Cancer Center, 651 Dongfeng East Road, Guangzhou, 510060 China; 3 Shantou University Medical College, Shantou University, Shantou, China; 4 Department of VIP Region, Sun Yat-Sen University Cancer Center, Guangzhou, China

**Keywords:** Biomarker, Immunotherapy, Chemoimmunotherapy, Serum amyloid A, Non-small cell lung cancer

## Abstract

Immune checkpoint inhibitors (ICIs) have transformed the treatment landscape for advanced non-small cell lung cancer (NSCLC), yet durable responses remain limited in a subset of patients. Serum amyloid A (SAA), an acute-phase protein linked to systemic inflammation, may reflect dynamic immune responses. This retrospective study analyzed 242 advanced NSCLC patients treated with first-line chemoimmunotherapy or immunotherapy between August 2016 and December 2024. Patients were stratified by early SAA kinetics into flare-responders (initial rise followed by decline), responders (sustained decline), and non-responders. Clinical outcomes, including progression-free survival (PFS) and overall survival (OS), were evaluated using Kaplan–Meier and Cox regression analyses. In the chemoimmunotherapy cohort, SAA flare-responders demonstrated significantly prolonged median PFS (29.8 months, 95% CI: 9.95—49.65; HR: 0.31, 95% CI: 0.15–0.64; *p* < 0.01) compared to non-responders (7.4 months, 95% CI: 4.67—10.13). Similarly, in the immunotherapy cohort, SAA flare-responders showed superior PFS. (19.9 vs. 2.1 months, HR 0.31, *p* < 0.01). Multivariate analysis confirmed early SAA kinetics as an independent prognostic factor for both PFS and OS in both treatment groups. Early SAA kinetics serve as a promising non-invasive biomarker for predicting clinical outcomes in advanced NSCLC treated with first-line chemoimmunotherapy or immunotherapy. These findings highlight SAA kinetics as a potential non-invasive biomarker and monitoring SAA dynamics may aid in identifying patients with higher likelihood of clinical benefit; however, prospective studies are required to determine its utility in guiding therapeutic decisions.

**Supplementary Information:**

The online version contains supplementary material available at 10.1186/s40364-025-00791-1.

## To the editor:

Immune checkpoint inhibitors (ICIs) targeting the programmed cell death 1(PD-1)/programmed cell death 1 ligand (PD-L1) axis have revolutionized the treatment of advanced non-small cell lung cancer (NSCLC). ICIs alone or combined with platinum-based chemotherapy (hereafter termed chemoimmunotherapy) are now standard first-line therapies for advanced NSCLC without driver mutations [1]. Although compelling clinical responses have been observed in various tumor types, only a small proportion of patients experience long-lasting benefits. Identifying biomarkers to predict clinical benefit remains critical for optimizing treatment strategies [2].

Systemic inflammation, a key driver of tumorigenesis and immune suppression, shapes the tumor microenvironment and influences therapeutic outcomes [3]. Serum amyloid A (SAA), an acute-phase protein elevated during inflammation, has shown correlations with PD-(L)1 inhibitor efficacy [4, 5]. However, its predictive role in chemoimmunotherapy—where inflammation dynamics may differ—remains undefined.

The"flare"kinetic pattern defined by Fukuda et al. [6], characterized by an initial biomarker surge followed by a decline below baseline, reflects dynamic immune-inflammatory interactions during therapy. Here, we investigate whether early SAA kinetics—flare response, sustained response, or non-response—predict survival outcomes in advanced NSCLC patients undergoing first-line chemoimmunotherapy or immunotherapy.

**Fig. 1:**
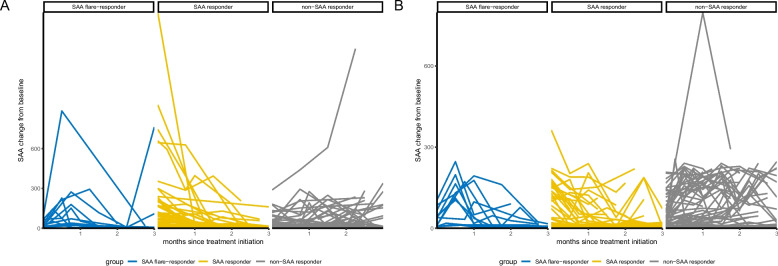
The time-series behaviors of SAA after treatment initiation in 12 weeks. **A**, chemoimmunotherapy cohort; **B**, immunotherapy cohort. SAA, serum amyloid A

In this retrospective analysis, we evaluated 242 advanced NSCLC patients without sensitizing genetic alterations treated with PD-(L)1 inhibitor monotherapy or first-line chemoimmunotherapy between August 2016 and December 2024. Patients were categorized into flare-responders, responders, and non-responders based on early SAA kinetics (Figure S1). The detailed comparison of baseline characteristics between patients in different groups was summarized in Table 1. The SAA variation in different time points after monotherapy or combination therapy initiation in all patients was shown in Fig. 1.

**Table 1:** Comparison of baseline characteristics of patients in SAA groups

Characteristics		Chemoimmunotherapy n (%)				*P* value	Immunotherapy n (%)				*P* value
		Total cohort	SAA flare- responder	SAA responder	Non-SAA responder		Total cohort	SAA flare-responder	SAA responder	Non-SAA responder	
No. of patients		132	13 (10)	60 (45)	59 (45)	-	110	12 (11)	39 (35)	59 (54)	-
Age, years	Median (range)	61 (53)	61 (31)	61 (42)	61 (53)	0.84	59 (51)	61 (39)	55 (39)	60 (49)	0.22
Gender	Male	107 (81)	12 (92)	53 (88)	42 (71)	0.03	81 (74)	9 (75)	30 (77)	42 (71)	0.81
	Female	25 (19)	1 (8)	7 (12)	17 (29)		29 (26)	3 (25)	9 (23)	17 (29)	
ECOG PS	0	27 (20)	2 (15)	13 (22)	12 (20)	1.00	39 (35)	4 (33)	17 (44)	18 (31)	0.52
	1	96 (73)	10 (77)	43 (72)	43 (73)		67 (61)	7 (58)	21 (54)	39 (66)	
	2	9 (7)	1 (8)	4 (7)	4 (7)		4 (4)	1 (8)	1 (3)	2 (3)	
Smoking history	No	57 (43)	5 (38)	22 (37)	30 (51)	0.28	60 (55)	8 (67)	19 (49)	33 (56)	0.52
	Yes	75 (57)	8 (62)	38 (63)	29 (49)		50 (45)	4 (33)	20 (51)	26 (44)	
PD-L1 level	+	22 (17)	3 (23)	10 (17)	9 (15)	0.49	4 (4)	2 (17)	1 (3)	1 (2)	0.08
	-	16 (12)	0 (0)	6 (10)	10 (17)		1 (1)	0 (0)	1 (3)	0 (0)	
	NA	94 (71)	10 (77)	44 (73)	40 (68)		105 (95)	10 (83)	37 (95)	58 (98)	
Histology	Adenocarcinoma	70 (53)	9 (69)	25 (42)	36 (61)	0.19	60 (55)	8 (67)	19 (49)	33 (56)	0.58
	Squamous	47 (36)	3 (23)	27 (45)	17 (29)		45 (41)	3 (25)	18 (46)	24 (41)	
	Other	15 (11)	1 (8)	8 (13)	6 (10)		5 (5)	1 (8)	2 (5)	2 (3)	
Stage	IIIB-IIIC	19 (14)	2 (15)	8 (13)	9 (15)	0.95	8 (7)	1 (8)	3 (8)	4 (7)	1.00
	IV	113 (86)	11 (85)	52 (87)	50 (85)		102 (93)	11 (92)	36 (92)	55 (93)	
Sites of metastases	Liver	21 (16)	1 (8)	13 (22)	7 (12)	0.24	25 (23)	2 (17)	8 (21)	15 (25)	0.74
	Lung	34 (26)	6 (46)	13 (22)	15 (25)	0.19	49 (45)	4 (33)	17 (44)	28 (47)	0.66
	Bone	35 (27)	2 (15)	17 (28)	16 (27)	0.63	39 (35)	4 (33)	13 (33)	22 (37)	0.91
	Brain	27 (20)	2 (15)	13 (22)	12 (20)	0.88	22 (20)	3 (25)	7 (18)	12 (20)	0.86
Lines of treatment	1	132 (100)	13 (100)	60 (100)	59 (100)	-	12 (11)	1 (8)	6 (15)	5 (8)	0.54
	≥ 2	0 (0)	0 (0)	0 (0)	0 (0)		98 (89)	11 (92)	33 (85)	54 (92)	
Baseline NLR	Median (range)	3.27 (18.77)	3.03 (9.24)	3.40 (18.61)	2.91 (8.23)	0.16	3.29 (36.03)	3.09 (5.48)	4.00 (36.00)	2.88 (16.04)	0.05
Baseline LDH, (IU/L)	Median (range)	208.10 (960.30)	216.90 (221.60)	203.20 (734.10)	215.10 (940.60)	0.36	159.07 (1065.60)	203.00 (426.40)	213.00 (1063.40)	203.40 (645.30)	0.76
Baseline albumin, (g/L)	Median (range)	42.00 (45.36)	42.40 (16.00)	40.35 (45.36)	43.30 (14.80)	< 0.01	42.05 (20.60)	42.75 (10.70)	41.30 (20.60)	42.50 (19.10)	0.22
Baseline CRP,(mg/L)	Median (range)	10.66 (184.34)	4.38 (124.01)	27.37 (182.16)	3.46 (151.02)	< 0.01	13.64 (167.98)	7.69 (43.05)	23.90 (124.53)	9.65 (167.97)	0.21
Baseline SAA,(mg/L)	Median (range)	23.95 (1625.50)	13.20 (87.10)	97.30 (1619.70)	9.90 (288.80)	< 0.01	34.90 (361.80)	38.00 (98.20)	83.70 (359.90)	18.80 (206.50)	0.02

*Abbreviations*: *ECOG PS* Eastern Cooperative Oncology Group performance status, *NLR* neutrophil-to-lymphocyte ratio, *LDH* lactate dehydrogenase, *CRP* C-reactive protein, *SAA* Serum amyloid A

**Fig. 2:**
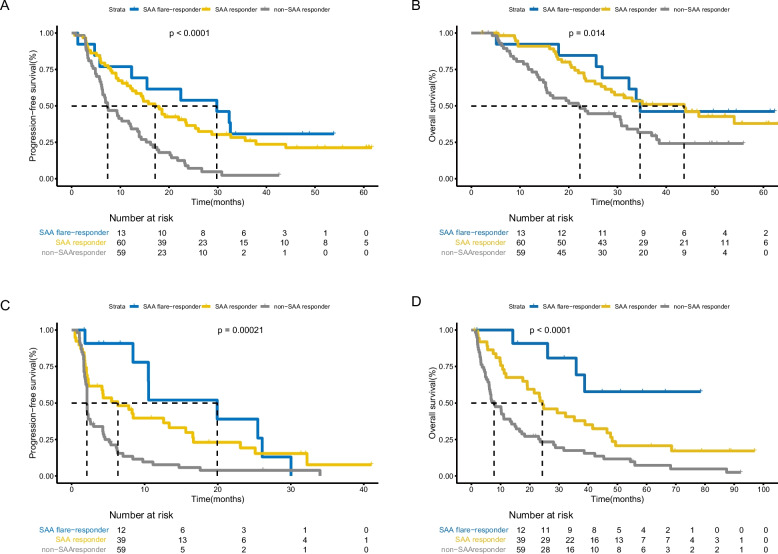
**A** Progression-free survival curves and (**B**) Overall survival curves based on early SAA kinetics in chemoimmunotherapy cohort. **C** Progression-free survival curves and (**D**) Overall survival curves based on early SAA kinetics in immunotherapy cohort. SAA, serum amyloid A

The key findings were striking: In the chemoimmunotherapy cohort, the group with SAA flare-responder, SAA-responder, and non-SAA responder had an ORR of 46% (6 of 13), 63% (38 of 60) and 34% (20 of 59), respectively (*p* = 0.01). The flare-responder group exhibited a median PFS of 29.8 months (95% CI 9.95–49.65, HR 0.31, 95% CI 0.15–0.64, *p* < 0.01), significantly longer than non-responders (7.4 months, 95% CI 4.67–10.13; Fig. 2A). Similarly, in the immunotherapy cohort, the group with SAA flare-responder, SAA-responder, and non-SAA responder had an ORR rate of 25% (3 of 12), 21% (8 of 39) and 7% (4 of 59), respectively (*p* = 0.07). SAA flare-responders had a median PFS of 19.9 months (95% CI: 7.54–32.32; HR: 0.31, 95% CI: 0.15–0.66; *p* < 0.01), compared to 2.1 months (95% CI: 2.05–2.15) in non-responders (Fig. 2C). In multivariate cox regression analysis about chemoimmunotherapy, we confirmed that early SAA kinetics was the only predictive factor associated with PFS, after adjusting for potential confounders such as age, gender and Eastern Cooperative Oncology Group performance status (ECOG PS) etc. (Table 2). Early SAA kinetics along with baseline lactate dehydrogenase (LDH) and neutrophil-to- lymphocyte ratio (NLR) group were still independently associated with OS (Table S1).

**Table 2:** Univariate and multivariate Cox regression analysis regarding progression-free survival (PFS)

Variables		Chemoimmunotherapy				Immunotherapy			
		Univariate		Multivariate		Univariate		Multivariate	
		HR (95%CI)	*P* value	HR (95%CI)	*P* value	HR (95%CI)	*P* value	HR (95%CI)	*P* value
Age	Continuous	0.98 (0.96–1.00)	**0.03**	0.99 (0.97–1.00)	0.09	1.00 (0.98–1.02)	0.59		
Gender	Male	0.89 (0.54–1.47)	0.66			0.74 (0.47–1.15)	0.18		
	Female	Ref				Ref			
ECOG PS	0	1.10 (0.47–2.60)	0.82			0.91 (0.32–2.58)	0.86		
	1	1.05 (0.49–2.29)	0.89			1.01 (0.36–2.79)	0.99		
	2	Ref				Ref			
Smoking History	Yes	0.86 (0.58–1.26)	0.43			0.92 (0.61–1.38)	0.67		
	No	Ref				Ref			
Histology	Adenocarcinoma	0.90 (0.47–1.73)	0.76			1.75 (0.55–5.61)	0.35		
	Squamous	0.97 (0.50–1.90)	0.94			1.57 (0.48–5.10)	0.45		
	Other	Ref				Ref			
Stage	IV	1.03 (0.60–1.75)	0.92			1.92 (0.70–5.25)	0.21		
	IIIB-IIIC	Ref				Ref			
Liver Metastasis	Yes	1.06 (0.61–1.77)	0.82			1.79 (0.11–2.88)	**0.02**	2.24 (1.34–3.75)	** < 0.01**
	No	Ref				Ref			
Lung Metastasis	Yes	1.28 (0.83–1.97)	0.27			1.05 (0.70–1.57)	0.82		
	No	Ref				Ref			
Bone Metastasis	Yes	1.22 (0.79–1.87)	0.37			1.20 (0.78–1.84)	0.41		
	No	Ref				Ref			
Brain Metastasis	Yes	1.03 (0.65–1.61)	0.91			1.51 (0.91–2.50)	0.11		
	No	Ref				Ref			
PD-L1 Level	+	1.03 (0.63–1.69)	0.91			0.83 (0.30–2.27)	0.72		
	-/NA	Ref				Ref			
Baseline NLR	Continuous	1.01 (0.94–1.09)	0.76			1.04 (0.99–1.09)	0.10		
	≤ 3.33	0.81 (0.55–1.18)	0.27			0.69 (0.46–1.03)	0.07		
	> 3.33	Ref				Ref			
Baseline LDH, (IU/L)	Continuous	1.00 (1.00–1.00)	0.05			1.00 (1.00–1.00)	0.28		
	≤ 250	1.05 (0.69–1.59)	0.81			0.70 (0.46–1.07)	0.10		
	> 250	Ref				Ref			
Baseline albumin, (g/L)	Continuous	1.04 (1.00–1.09)	**0.04**	1.01 (0.98–1.05)	0.50	0.93 (0.88–0.99)	**0.01**	0.97 (0.91–1.04)	0.45
Baseline CRP, (mg/L)	Continuous	1.00 (1.00–1.01)	0.86			1.01 (1.00–1.01)	** < 0.01**	1.01 (1.00–1.02)	0.30
	≤ 10	0.99 (0.68–1.45)	0.96			0.66 (0.43–0.99)	**0.05**	0.80 (0.44–1.47)	0.48
	> 10	Ref				Ref		Ref	
Baseline SAA, (mg/L)	Continuous	1.00 (1.00–1.00)	0.58			1.00 (1.00–1.01)	** < 0.01**	1.00 (1.00–1.01)	**0.02**
Early SAA Kinetics	SAA Flare-Responder	0.31 (0.15–0.64)	** < 0.01**	0.34 (0.16–0.70)	** < 0.01**	0.31 (0.15–0.66)	** < 0.01**	0.23 (0.10–0.51)	** < 0.01**
	SAA Responder	0.42 (0.28–0.64)	** < 0.01**	0.45 (0.29–0.69)	** < 0.01**	0.48 (0.30–0.75)	** < 0.01**	0.32 (0.19–0.54)	** < 0.01**
	Non-SAA Responder	Ref				Ref		Ref	

*Abbreviations*: *ECOG PS* Eastern Cooperative Oncology Group performance status, *NLR* neutrophil-to- lymphocyte ratio, *LDH* lactate dehydrogenase, *CRP* C-reactive protein, *SAA* Serum amyloid A

These results are particularly significant given the current challenges in predicting response to ICIs and chemoimmunotherapy in advanced NSCLC. While tissue-based biomarkers like PD-L1 expression and tumor mutational burden are commonly used [7, 8], they have limitations due to tumor heterogeneity and the invasiveness of biopsies. Recent efforts have focused on developing blood-based biomarkers to predict patient response to ICIs, complementing tumor-based biomarkers. Blood-based approaches such as circulating levels of cytokines and other soluble factors, exosomes, or circulating tumor DNA (ctDNA) offer non-invasive, dynamic monitoring advantages. The dynamic monitoring of SAA levels offers a less invasive approach and provides real-time insights into the systemic inflammatory response, which is closely linked to the tumor microenvironment and treatment efficacy.

The concept of"flare-response"kinetics, initially described by Fukuda et al. in metastatic renal cell carcinoma [6], appears to be a promising indicator of early immune system activation in NSCLC as well, due to shared systemic inflammatory mechanisms and emerging evidence linking acute-phase proteins to ICIs efficacy across cancers. The initial rise in SAA levels, followed by a subsequent drop below baseline, may reflect the dynamic phase of systemic inflammation induced by anti-tumor immune responses. This phenomenon could serve as an early marker for identifying patients who are likely to benefit from ICIs or chemoimmunotherapy, potentially guiding treatment decisions and improving patient outcomes.

Other serum indexes have also been proposed to be potential predictors of the effect of ICIs-based treatment. A low NLR and LDH level are associated with greater benefit from immunotherapy and first-line chemoimmunotherapy [9, 10]. Our findings are consistent with these results regarding the association between these serum biomarkers and treatment efficacy or prognosis. However, after adjusting the confounding factors, baseline NLR and LDH showed no predictive power for PFS in two treatment group, although baseline NLR was independently associated with OS in both monotherapy and combination therapy. While ctDNA reflects tumor burden reduction, its high cost and technical complexity limit accessibility. SAA, a low-cost, routine assay, offers dynamic insights into inflammation, complementing ctDNA’s molecular profiling. Moreover, compared with other single inflammatory markers and even baseline SAA, SAA kinetics can provide more information in predicting monotherapy or combination therapy.

However, our study has some limitations include: 1) Retrospective design and relative small sample size with potential selection bias; 2) Heterogeneity in treatment regimens (e.g., varying chemotherapy agents and ICI types); 3) Lack of standardized SAA measurement timepoints across cohorts, which may affect kinetic categorization. Future prospective studies with larger cohorts are needed to validate these findings and establish standardized definitions for SAA flare-response. Additionally, the biological mechanisms linking SAA dynamics to immune activation remain speculative and require mechanistic validation. Preclinical studies suggest SAA was involved in the modulation of tumor immunity, potentially impacting tumor progression and therapeutic resistance. For example, SAA could diminish the cytotoxic activity of T cells and contributes to resistance against PD-1 antibody by attracting neutrophils and enhancing their PD-L1 expression through the LDHA/STAT3 pathway [11]; Besides, SAA could restrain dendritic cells and anti-tumor T cell immunity through TLR2 signaling [12]. However further research is warranted to elucidate the biological mechanisms underlying the role of SAA kinetics in NSCLC and its potential interactions with other inflammatory markers.

In conclusion, our study provides evidence that early SAA kinetics may serve as a valuable prognostic biomarker in advanced NSCLC. This approach could enhance our ability to predict treatment response and tailor therapeutic strategies, ultimately improving the management of advanced NSCLC.

## Supplementary Information

Supplementary Material 1.

## Data Availability

No datasets were generated or analysed during the current study.
