# Supplementary material for: Early kinetics of serum amyloid A predict clinical benefit to first-line chemoimmunotherapy and immunotherapy in advanced non-small cell lung cancer: a retrospective analysis
Source: Biomark Res. 2025 May 24;13:76. doi: 10.1186/s40364-025-00791-1 (PMC12102904; doi:10.1186/s40364-025-00791-1)
Supplement: Supplementary file 1 — Supplementary Material 1. [file 40364_2025_791_MOESM1_ESM.docx]

supplementary material

**Figure S1 Diagram of three defined groups (flare-responders, responders, and non-responders) according to early SAA kinetics. SAA, serum amyloid A**

**
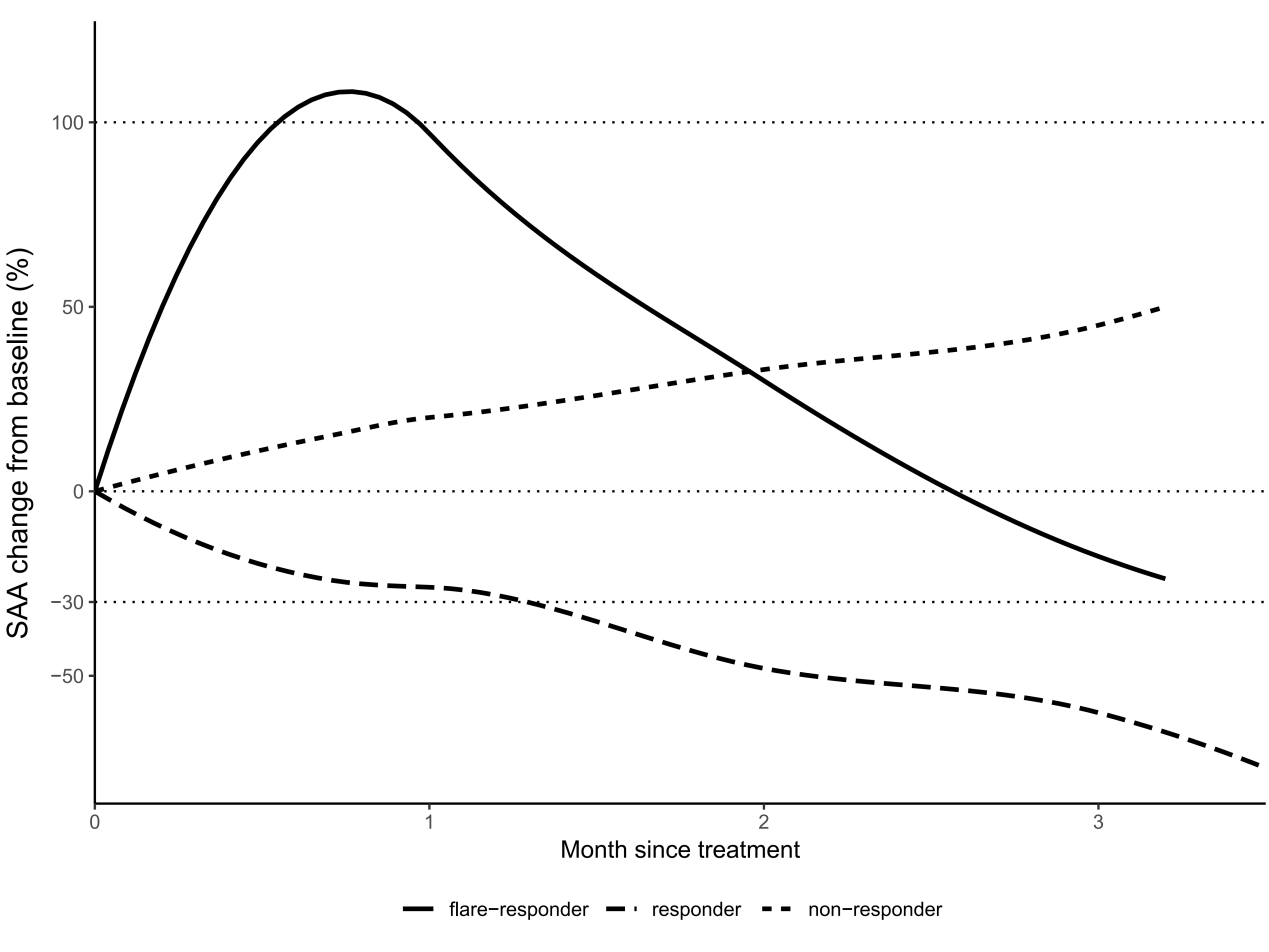
**

**Table S1** Comparison of baseline characteristics of patients in SAA groups.

| Characteristics | | Chemoimmunotherapy n (%) | | | | *P*  value | Immunotherapy n (%) | | | | *P*  value |
| --- | --- | --- | --- | --- | --- | --- | --- | --- | --- | --- | --- |
|  |  | Total  cohort | SAA flare-  responder | SAA  responder | Non-SAA  responder |  | Total  cohort | SAA flare-  responder | SAA  responder | Non-SAA  responder |  |
| No. of patients | | 132 | 13(10) | 60(45) | 59(45) | - | 110 | 12(11) | 39(35) | 59(54) | - |
| Age, years | Median (range) | 61(53) | 61(31) | 61(42) | 61(53) | 0.84 | 59(51) | 61(39) | 55(39) | 60(49) | 0.22 |
| Gender | Male | 107(81) | 12(92) | 53(88) | 42(71) | 0.03 | 81(74) | 9(75) | 30(77) | 42(71) | 0.81 |
|  | Female | 25(19) | 1(8) | 7(12) | 17(29) |  | 29(26) | 3(25) | 9(23) | 17(29) |  |
| ECOG  PS | 0 | 27(20) | 2(15) | 13(22) | 12(20) | 1.00 | 39(35) | 4(33) | 17(44) | 18(31) | 0.52 |
|  | 1 | 96(73) | 10(77) | 43(72) | 43(73) |  | 67(61) | 7(58) | 21(54) | 39(66) |  |
|  | 2 | 9(7) | 1(8) | 4(7) | 4(7) |  | 4(4) | 1(8) | 1(3) | 2(3) |  |
| Smoking  history | No | 57(43) | 5(38) | 22(37) | 30(51) | 0.28 | 60(55) | 8(67) | 19(49) | 33(56) | 0.52 |
|  | Yes | 75(57) | 8(62) | 38(63) | 29(49) |  | 50(45) | 4(33) | 20(51) | 26(44) |  |
| PD-L1  level | + | 22(17) | 3(23) | 10(17) | 9(15) | 0.49 | 4(4) | 2(17) | 1(3) | 1(2) | 0.08 |
|  | - | 16(12) | 0(0) | 6(10) | 10(17) |  | 1(1) | 0(0) | 1(3) | 0(0) |  |
|  | NA | 94(71) | 10(77) | 44(73) | 40(68) |  | 105(95) | 10(83) | 37(95) | 58(98) |  |
| Histology | Adenocarcinoma | 70(53) | 9(69) | 25(42) | 36(61) | 0.19 | 60(55) | 8(67) | 19(49) | 33(56) | 0.58 |
|  | Squamous | 47(36) | 3(23) | 27(45) | 17(29) |  | 45(41) | 3(25) | 18(46) | 24(41) |  |
|  | Other | 15(11) | 1(8) | 8(13) | 6(10) |  | 5(5) | 1(8) | 2(5) | 2(3) |  |
| Stage | IIIB-IIIC | 19(14) | 2(15) | 8(13) | 9(15) | 0.95 | 8(7) | 1(8) | 3(8) | 4(7) | 1.00 |
|  | IV | 113(86) | 11(85) | 52(87) | 50(85) |  | 102(93) | 11(92) | 36(92) | 55(93) |  |
| Sites of  metastases | Liver | 21(16) | 1(8) | 13(22) | 7(12) | 0.24 | 25(23) | 2(17) | 8(21) | 15(25) | 0.74 |
|  | Lung | 34(26) | 6(46) | 13(22) | 15(25) | 0.19 | 49(45) | 4(33) | 17(44) | 28(47) | 0.66 |
|  | Bone | 35(27) | 2(15) | 17(28) | 16(27) | 0.63 | 39(35) | 4(33) | 13(33) | 22(37) | 0.91 |
|  | Brain | 27(20) | 2(15) | 13(22) | 12(20) | 0.88 | 22(20) | 3(25) | 7(18) | 12(20) | 0.86 |
| Lines of  treatment | 1 | 132(100) | 13(100) | 60(100) | 59(100) | - | 12(11) | 1(8) | 6(15) | 5(8) | 0.54 |
|  | ≥2 | 0(0) | 0(0) | 0(0) | 0(0) |  | 98(89) | 11(92) | 33(85) | 54(92) |  |
| Baseline NLR | Median  (range) | 3.27  (18.77) | 3.03  (9.24) | 3.40  (18.61) | 2.91  (8.23) | 0.16 | 3.29  (36.03) | 3.09  (5.48) | 4.00  (36.00) | 2.88  (16.04) | 0.05 |
| Baseline  LDH, (IU/L) | Median  (range) | 208.10  (960.30) | 216.90  (221.60) | 203.20  (734.10) | 215.10  (940.60) | 0.36 | 159.07  (1065.60) | 203.00  (426.40) | 213.00  (1063.40) | 203.40  (645.30) | 0.76 |
| Baseline  albumin, (g/L) | Median  (range) | 42.00  (45.36) | 42.40  (16.00) | 40.35  (45.36) | 43.30  (14.80) | <0.01 | 42.05  (20.60) | 42.75  (10.70) | 41.30  (20.60) | 42.50  (19.10) | 0.22 |
| Baseline  CRP mg/L | Median  (range) | 10.66  (184.34) | 4.38  (124.01) | 27.37  (182.16) | 3.46  (151.02) | <0.01 | 13.64  (167.98) | 7.69  (43.05) | 23.90  (124.53) | 9.65  (167.97) | 0.21 |
| Baseline  SAA mg/L | Median  (range) | 23.95  (1625.50) | 13.20  (87.10) | 97.30  (1619.70) | 9.90  (288.80) | <0.01 | 34.90  (361.80) | 38.00  (98.20) | 83.70  (359.90) | 18.80  (206.50) | 0.02 |

Abbreviations: ECOG PS, Eastern Cooperative Oncology Group performance status; NLR, neutrophil-to-lymphocyte ratio; LDH, lactate dehydrogenase; CRP, C-reactive protein; SAA, Serum amyloid A.

**MATERIALS AND METHODS**

**Patients Selection**

**In this retrospective analysis, eligible patients were 18 years or older, diagnosed with metastatic or unresectable NSCLC who received ICIs or first-line ICIs in combination with platinum-doublet chemotherapy at Sun Yat-Sen University Cancer Center (SYSUCC) between August 2016 and December 2024. Patients lacking baseline or on-treatment circulating SAA data within 12 weeks were excluded from the final analysis. This study was approved by the ethics committee of Sun Yat-Sen University Cancer Center (approved number: B2020-402-01) with a waiver of informed consent since the data was deidentified. All procedures performed in this study were conducted following the Declaration of Helsinki (as revised in 2013).**

**Data Collection**

**Demographic and clinical variables**

**The following pretreatment demographic and clinical information was ascertained from our electronic healthcare database: age, gender, Eastern Cooperative Oncology Group performance status (ECOG PS), histology, stage, smoking history, sites of metastases, lines of treatment and PD-L1 level.**

**Definition of early SAA kinetics**

**The blood routine and biochemistry indexes were routinely detected by an automatic biochemical analyzer at baseline before the treatment initiation and before subsequent administration of ICIs or chemoimmunotherapy according to follow-up protocols. We recorded time-series-based serum SAA levels before (baseline) and during immunotherapy or chemoimmunotherapy for three months. According to the flare-kinetics definition (Figure 1) as previously described[1], flare-responders were defined as patients whose SAA levels rise to double their baseline level within 1 month (flare) and then drop to a lower level than baseline; responders were defined as patients whose SAA levels reduced by at least 30% without prior “flare”, and the rest was defined as non-responders.**

**Other Hematologic indexes**

**Moreover, other blood and biochemical indexes were also recorded including baseline absolute neutrophil count (ANC), baseline absolute lymphocyte count (ALC), baseline albumin and baseline lactate dehydrogenase (LDH). The NLR was defined as the ratio of ANC to ALC consistent with prior studies[2].**

**Endpoints**

**Tumor response evaluation for all patients was independently performed by two thoracic radiologists using computed tomography (CT) or magnetic resonance imaging (MRI) according to Response Evaluation Criteria in Solid Tumor (RECIST) v1.1. Patients underwent tumor assessment until treatment termination due to any reasons. The objective response rate (ORR) was defined as the proportion of patients**

**who had a complete response or partial response according to RECIST 1.1. The endpoints were progression-free survival (PFS), defined as the time elapsed from the start of treatment until progression, death, or censoring, and overall survival (OS), defined as the time elapsed from the start of treatment to death, with surviving patients censored at their date of the last follow-up.**

**Statistical Analysis**

**Continuous variables were expressed as median (range) and categorical data are depicted as numbers (percentages). Demographic and clinical data were compared by χ2 test or Fisher exact test for categorical variables and Mann-Whitney U test for continuous variables. For further statistical analyses, the following predetermined covariates were categorized based on clinically meaningful values and evaluated as candidate categorical variables for inclusion in the final cox proportional hazards models: lines of prior therapies (1vs. ≥ 2), PD-L1 level (positive vs negative vs NA), baseline LDH (high vs normal), baseline CRP (≤10 vs > 10), NLR (≤3.33 vs > 3.33)[2].**

**Survival curves (PFS and OS) were estimated using the Kaplan-Meier method and the intergroup survival differences were compared using a log-rank test. The potential predictors of survival were explored using a cox proportional hazards regression model. Characteristics with a *p* value < 0.05 after the univariate analysis were included in the multivariate analysis in a enter procedure. Both adjusted and unadjusted statistical methods were applied to estimate the hazard ratio (HR) and 95 % confidence interval (CI).**

**A two-sided, *p* < 0.05 was considered statistically significant. Statistical analyses were performed with SPSS version 26 and a graphical user interface for R (The R Foundation for Statistical Computing, Vienna, Austria).**

**Reference**

1. Fukuda, S., et al., *Impact of C-reactive protein flare-response on oncological outcomes in patients with metastatic renal cell carcinoma treated with nivolumab.* J Immunother Cancer, 2021. **9**(2).

2. Alessi, J.V., et al., *Clinicopathologic and Genomic Factors Impacting Efficacy of First-Line Chemoimmunotherapy in Advanced NSCLC.* J Thorac Oncol, 2023. **18**(6): p. 731-743.
